# A case of insulinoma with hypoglycemia that was better managed with lanreotide than octreotide

**DOI:** 10.1002/ccr3.4118

**Published:** 2021-05-19

**Authors:** Keiko Yamaoka, Shuichi Nagashima, Nobukazu Okada, Nagisa Sawayama, Shinsuke Saito, Manabu Takahashi, Kenta Okada, Kazuhiro Endo, Masaru Koizumi, Hideki Sasanuma, Ken Ebihara, Atsuko Kasajima, Noriyoshi Fukushima, Naohiro Sata, Shun Ishibashi

**Affiliations:** ^1^ Division of Endocrinology and Metabolism Department of Medicine Jichi Medical University Shimotsuke Japan; ^2^ Department of Surgery Jichi Medical University Shimotsuke Japan; ^3^ Department of Pathology Jichi Medical University Shimotsuke Japan; ^4^ Department of Pathology Tohoku University Graduate School of Medicine Sendai Japan; ^5^ Present address: Department of Pathology Technical University Munich Munich Germany

**Keywords:** hypoglycemia, insulinoma, lanreotide, octreotide, somatostatin analog

## Abstract

Long‐acting somatostatin analogs, including lanreotide slow release (LAN‐SR) and octreotide long‐acting release (OCT‐LAR), can improve hypoglycemia in insulinoma. LAN‐SR may be more beneficial in some patients with insulinoma than OCT‐LAR.

## Graphical abstract

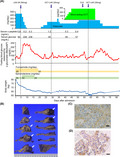

## 1 INTRODUCTION

Insulinoma is a pancreatic β cell tumor that causes hyperinsulinemic hypoglycemia. Although surgical resection is the primary treatment modality, medical treatments that alleviate hypoglycemia are essential for treatment of inoperable conditions, including advanced disease or tumors with unknown primary sites.^1^ Initial treatment for such patients includes the K_ATP_ channel opener, diazoxide, which inhibits the release of insulin from β cells,^2^ but may cause fluid retention.^3^ Somatostatin analogs (SSAs), which have fewer severe adverse side effects, can be used for the same purpose.^4^ Here, we report a patient with insulinoma who was treated with two SSAs, lanreotide slow release (LAN‐SR), and octreotide long‐acting release (OCT‐LAR), before tumor localization. These two agents caused different effects on blood glucose level and may have different hormonal efficacies.

## 2 CASE HISTORY

A 36‐year‐old Japanese man was admitted to our hospital for suspected insulinoma and an intolerance to diazoxide. The details of the patient's clinical course with respect to glycemic responses to diazoxide have been previously reported.^5^ In brief, at the age of 33, he exhibited hypoglycemia (46 mg/dL) and a low HbA1c level (3.4%; reference range: 4.6%‐6.2%) with unusually high levels of serum insulin (11.1 μU/mL) and C‐peptide (2.6 ng/dL). The patient's past medical history was significant for cerebral palsy with intellectual disability, which was diagnosed at the age of 3 years. Because of the patient's inability to understand instructions, we did not perform procedures requiring his cooperation, such as esophageal ultrasonography or intra‐arterial calcium‐stimulated venous sampling, for which patients were not routinely sedated in our institute. Instead, we initially performed less demanding abdominal ultrasonography and contrast‐enhanced computed tomography (CT), which were not sensitive enough to detect the tumor. Therefore, we initiated diazoxide treatment at doses titrated up to 150 mg/day to avoid hypoglycemia.^5^

After 3 years, the patient developed refractory hypoglycemia, requiring higher doses of diazoxide (max, 450 mg/day), as well as chronic side effects, including systemic edema and pleural effusion. He was subsequently admitted to our hospital for treatment reevaluation and lesion relocalization for surgical resection.

### 2.1 Differential diagnosis, investigation, and treatment

Endogenous hyperinsulinemic hypoglycemia may be present, when the laboratory data fulfill the following conditions: blood glucose <55 mg/dL, insulin >3 μU/mL, and C‐peptide >0.6 ng/dL.^6^ There was no history of prior exposure to any oral hypoglycemic agent, any gastric surgery, or insulin autoimmune syndrome, which causes endogenous hyperinsulinemic hypoglycemia. These findings suggested the possibility of insulinoma or noninsulinoma pancreatogenous hypoglycemic syndrome. During the detailed laboratory and imaging investigations, SSA treatment for hypoglycemia was initiated. Additionally, the efficacies of two long‐acting SSAs, LAN‐SR, and OCT‐LAR, which have different pharmacokinetic profiles,^7^ were compared. After subcutaneous injection, the plasma concentrations of LAN‐SR show an initial sharp increase that peaks on day 1, followed by a consistent decrease. In contrast, time course of plasma concentrations of OCT‐LAR consists of 3 distinct phases: a transient increase on day 1 after administration, followed by a progressive decline from day 2 to 6, then a slow increase after day 6‐8 with maintenance of a concentration plateau for about 14‐30 days, followed by a steady decrease.^7^

**FIGURE 1:**
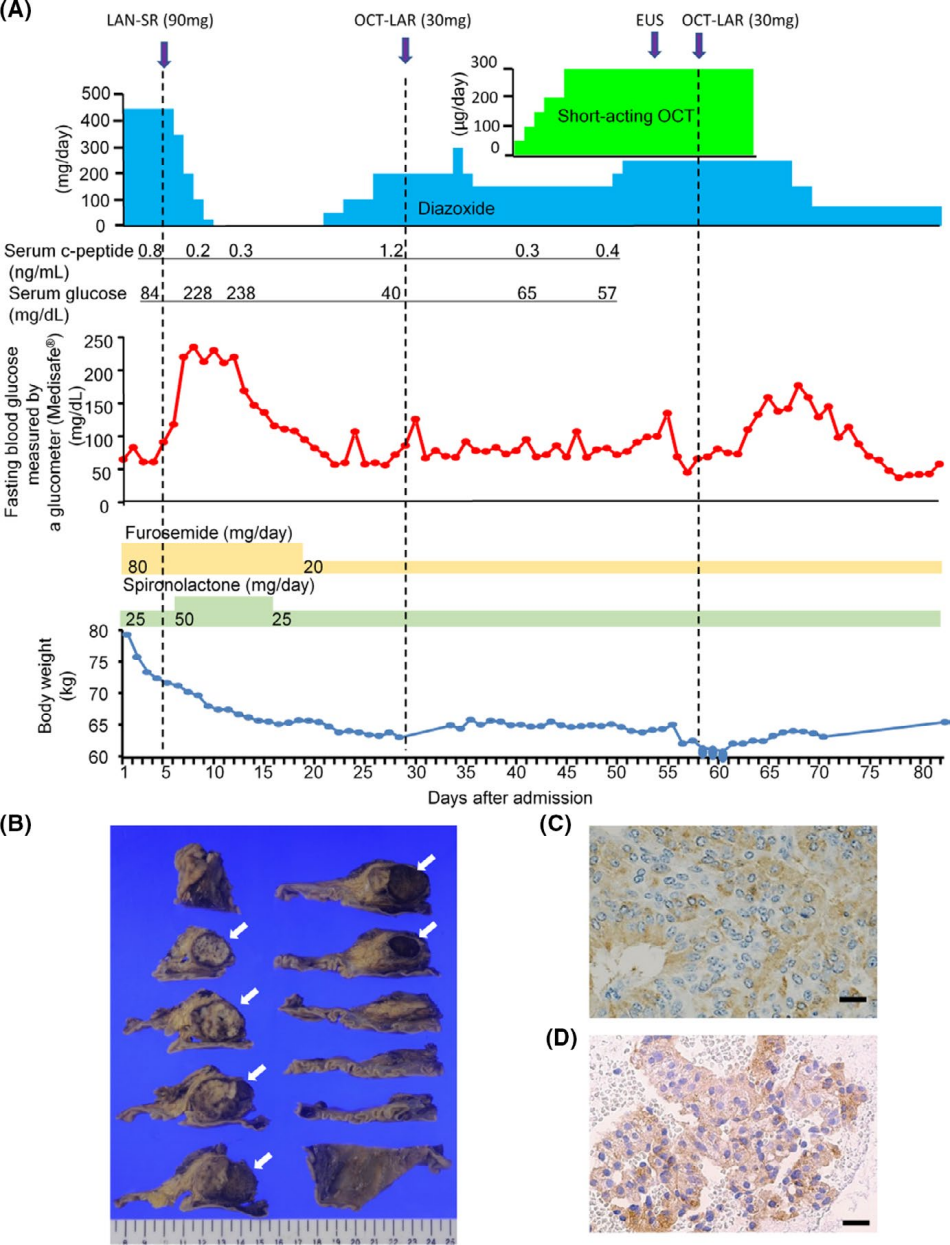
A, Clinical course of the patient treated with lanreotide slow release (LAN‐SR) and octreotide long‐acting release (OCT‐LAR). The hormonal treatment and examination are presented in the upper panel. The fasting blood glucose levels are presented in the middle panel. The diuretic treatment and body weight change are presented in the lower panel. The serum C‐peptide levels are described between the upper and middle panels. EUS, endoscopic ultrasonography. B, Surgical specimen: the head of the pancreas. The tumor (represented by arrowheads) was well‐circumscribed and measured approximately 3.4 cm × 3.2 cm × 2.0 cm in size. Representative immunohistochemical images of the tumor. Cytoplasmic expression of insulin (C) and focal membranous expression of somatostatin receptor 2 (D). Scale bar, 20 μm

Figure 1A shows the patient's clinical course before and after SSA administration. Initially, diazoxide was continued at a dose of 450 mg/day, and LAN‐SR (90 mg) was administered. After 3 days, the patient's fasting glucose level increased to >200 mg/dL. The diazoxide dose was reduced and eventually discontinued. In combination with the use of diuretics, the tapering of diazoxide caused a significant reduction in body weight, primarily reflecting the alleviation of fluid retention, which is a common adverse side effect of diazoxide. Fourteen days after lanreotide injection, the patient's fasting blood glucose decreased below 70 mg/dL. Diazoxide treatment was restarted prior to administration of OCT‐LAR (30 mg), because the plasma levels of OCT typically increase slowly. However, the increase in the blood glucose level was not sufficient to discontinue diazoxide. Since it was assumed the plasma concentration of octreotide did not reach a therapeutically effective level, short‐acting OCT was additionally administered, followed by a second dose of OCT‐LAR. Again, the efficacy of OCT was insufficient to discontinue diazoxide. Nineteen days after the second dose of OCT‐LAR, the patient's blood glucose level decreased below 40 mg/dL, which suggested a paradoxical worsening of hypoglycemia. Therefore, OCT therapy was discontinued.

Serum C‐peptide levels were reduced both by LAN‐SR (from 0.8 to 0.2 mg/dL) and the first administration of OCT‐LAR (from 1.2 to 0.3 mg/dL, Figure 1A). These observations indicated that OCT‐LAR and LAN‐SR suppressed intrinsic insulin secretion to the same degree.

Before the second administration of OCT‐LAR, we obtained a consent to perform invasive procedures from the patient's parents and successfully performed endoscopic ultrasonography under general anesthesia to identify a tumor located in the head of pancreas.

A month after tumor localization, a pylorus‐preserving pancreatoduodenectomy was performed. The tumor was macroscopically surgically resected. The tumor measured 3.4 cm, was sharply demarcated, and presented with a solid yellow‐brownish cut surface (Figure 1B). Microscopically, the tumor demonstrated trabecular architecture and was positive for insulin (Figure 1C). Somatostatin receptor (SSTR) 2 was membranous and focally cytoplasmic (Figure 1D), while SSTR5 was not expressed (data not shown). Two % of the tumor cells were positive for Ki‐67, and <2 mitoses were found per 10 HPF (data not shown). No metastatic lesions were found. The tumor was classified as an insulinoma of neuroendocrine tumor grade 1 (WHO 2017).^8^

### 2.2 Outcome and follow‐up

After surgery, the patient's blood glucose levels remain within a normal range without any medical interventions; all drugs were subsequently discontinued.

## 3 DISCUSSION

Here, we describe a patient with insulinoma who had different glycemic responses to LAN‐SR and OCT‐LAR. Hypoglycemia was significantly improved by LAN‐SR, but not by OCT‐LAR. Because both SSAs led to a sufficient reduction in serum C‐peptide, we speculate that the counter‐regulatory hormones for insulin were more significantly suppressed by OCT‐LAR than by LAN‐SR. To our knowledge, this is the first report to describe the different efficacies of these SSAs in an insulinoma patient.

The anti‐secretory effects of somatostatin on various hormones from endocrine organs may be predominantly regulated by SSTR2 and SSTR5.^9^ Although LAN‐SR and OCT‐LAR exhibit different pharmacokinetics,^7^ both SSAs have high affinities for these receptors.^9^

Octreotide is effective in patients with insulinoma, and this efficacy is associated with tumoral expression of SSTR2, but not SSTR5.^4^ The patient's tumor expressed SSTR2 exclusively at the time of surgery. Theoretically, OCT should also inhibit insulin secretion by binding to SSTR2 with a similar affinity to LAN‐SR; however, we observed significant differences in blood glucose responses, even though serum C‐peptide was almost equally decreased by OCT‐LAR and LAN‐SR.

Somatostatin analogs also inhibit the counter‐regulatory hormones glucagon and growth hormone (GH), which antagonize the effects of insulin. OCT has been reported to paradoxically aggravate hypoglycemia in some patients with insulinoma,^10^, ^11^, ^12^ which can be explained by the preferential stronger reduction in GH and/or glucagon secretion than insulin secretion.^10^, ^11^ There are only 4 reported cases of insulinoma treated with LAN‐SR.^13^, ^14^, ^15^, ^16^ Two cases were benign, and the other 2 were malignant insulinoma with liver metastasis. In the benign cases, hypoglycemia improved after treatment,^13^, ^14^ but the hypoglycemia did not alleviate in the malignant cases.^15^, ^16^ In these 4 cases, paradoxical hypoglycemia was not observed.

Lanreotide and OCT inhibit GH and glucagon secretion; however, differences in the efficacies of LAN and OCT have been reported. In a prospective crossover study comparing the efficacy of LAN‐SR and OCT‐LAR in 10 patients with acromegaly, OCT‐LAR decreased GH levels 12% more than LAN‐SR after 6 months of treatment.^17^ Furthermore, in healthy volunteers, LAN did not affect plasma glucagon levels, although it decreased the plasma levels of insulin and insulin‐like growth factor (IGF)‐1.^18^ Moreover, a treatment change from LAN‐SR to OCT‐LAR has been reported to decrease insulin requirements in a diabetic patient with acromegaly^19^; a decrease in plasma glucagon without affecting GH, IGF‐1, and insulin levels following OCT‐LAR administration was also observed. In this case, we speculated that LAN‐SR might inhibit the secretion of these counter‐regulatory hormones to a lesser degree than OCT‐LAR; however, the precise mechanisms are unknown.

There are some limitations to this report. First, we did not measure circulating GH, IGF‐1, or glucagon levels, which would be necessary to prove our hypothesis. Second, we did not perform a crossover comparison between the two drugs with a longer observation period, because the tumor was successfully identified for surgical resection. Third, we cannot rule out the possibility that LAN and OCT might inhibit the secretion of gastrointestinal hormones and exert different effects on glycemic control by differentially affecting nutrient absorption.

In conclusion, the effects of LAN‐SR and OCT‐LAR on blood glucose levels in hypoglycemic patients with insulinoma may differ. LAN‐SR may be more useful than OCT‐LAR in alleviating hypoglycemia that is refractory to traditional medical therapy in patients with insulinoma.

## CONFLICT OF INTEREST

The authors declare no conflicts of interest.

## AUTHOR CONTRIBUTIONS

KY and SN: contributed to the patient's clinical care and preparation of the manuscript. NO, NSaw, SS, MT, KO, and KEb: contributed to the patient's clinical care and revision of the manuscript. KEn, MK, HS, and NSat: contributed to patient's surgery and revision of the manuscript. AK and NF: contributed to the histopathological assessment and revision of the manuscript. SI: contributed to the patient's clinical care, revision, and final approval of the manuscript of the manuscript. All authors approved the final manuscript.

## PATIENT CONCENT

Because of the patient's inability to understand instructions due to cerebral palsy, written informed consent was obtained from the patient's parents for publication of this case reports and any accompanying images.

## Data Availability

The data that support the findings of this study are available from the corresponding author upon reasonable request.
